# Impact of pharmacist counseling on health-related quality of life of patients with type 2 diabetes mellitus: a cluster randomized controlled study

**DOI:** 10.1007/s40200-020-00528-x

**Published:** 2020-06-03

**Authors:** Fajriansyah, Aulia Iskandarsyah, Irma M Puspitasari, Keri Lestari

**Affiliations:** 1 Department of Pharmacology and Clinical Pharmacy, Faculty of Pharmacy, Universitas Padjadjaran, Jl. Raya Bandung Sumedang KM 21, Jatinangor, West Java 45363 Indonesia; 2 Sekolah Tinggi Ilmu Farmasi Makassar, South Sulawesi, Indonesia; 3 Department of Clinical Psychology, Faculty of Psychology, Universitas Padjadjaran, Jatinangor-Sumedang, West Java Indonesia; 4 Centre of Excellence for Pharmaceutical Care Innovation, Universitas Padjadjaran, Jatinangor-Sumedang, West Java Indonesia

**Keywords:** HRQoL, Prolanis, Type 2 diabetes mellitus, Pharmacist counseling, EQ-5D-5 L

## Abstract

**Purpose:**

The quality of life (QoL) of patients with type 2 diabetes mellitus (T2DM) is a measure of the successful outcomes of therapy. The program of management of chronic diseases “Program Pengelolaan Penyakit Kronis” (Prolanis) among patients with hypertension and T2DM is a new strategy of the Badan Penyelenggara Jaminan Sosial (BPJS), which is the Indonesian national health insurance system. Here, we analyzed the impact of pharmacist counseling interventions on health-related QoL (HRQoL) in Prolanis T2DM patients.

**Methods:**

This cluster randomized controlled trial was designed to include two groups [control (*n* = 111) and intervention (*n* = 109) groups], and pre- and post-test procedures. The participants were Prolanis T2DM patients who attended four primary health-care centers (Puskesmas) in Makassar City, South Sulawesi, Indonesia from August 2017 to August 2018. The intervention group received systematic counseling for 6 months. The data were collected using the Bahasa Indonesia version of the European Quality of Life 5 Dimensions 5 Levels (EQ-5D-5 L) questionnaire and were analyzed using EQ-5D preference weight for each health state with the Indonesian EQ-5D-5 L value Set. Furthermore, the EQ-5D index and the EQ-5D VAS score were calculated and HbA1c levels were assessed.

**Results:**

The change in the EQ-5D-5 L index score (post-pre) was 0.01 in the control group and 0.04 in the intervention group (*P* = 0.041). The change in the VAS score was −0.07in the control group (post-pre) and 2.66 in the intervention group (*P* = 0.000).

**Conclusion:**

Pharmacist counseling may help improve the HRQoL of Prolanis T2DM patients.

**Electronic supplementary material:**

The online version of this article (10.1007/s40200-020-00528-x) contains supplementary material, which is available to authorized users.

## Introduction

The number of people with diabetes worldwide is expected to increase to 592 million in 2035 [1]. Indonesia faces a situation that is similar to the global threat of diabetes. The International Diabetes Federation (IDF) reports that the diabetes epidemic in Indonesia is still showing an increasing trend [2]. Indonesia is the sixth-ranked country in the world, after China, India, the United States, Brazil, and Mexico, regarding the number of people with diabetes aged 20–79 years (~10.3 million people) [2]. Accordingly, the Basic Health Research (RISKESDAS), Ministry of Health, Indonesia reports a significant increase in the prevalence of diabetes, from 6.9% in 2013 to 8.5% in 2018. In addition, this agency estimated that more than 16 million people have diabetes mellitus, especially type 2 diabetes mellitus (T2DM), in Indonesia [3].

Several randomized clinical trials described the effect of various factors on the quality of life (QoL) of patients diagnosed with T2DM [4] [5]. Health-related quality of life (HRQoL) is an individual’s assessment of the welfare conditions related to health [6]. Moreover, according to the report of the Institute of Health Economics (IHE) of 2008, QoL is a health status that is approved by the subjective perception of patients or individuals [7]. The assessment of the QoL can determine the compatibility between groups or between individuals at one point in time, measure changes in individuals or groups over a specific period, and predict a future situation [7].

The Badan Penyelenggara Jaminan Sosial (BPJS) is an authorized body that was established to provide a medical coverage program for the Indonesian people [8]. The BPJS, which focuses on health insurance, started its operation in 2014 [8]. One of the new strategies of the BPJS is the program of management of chronic diseases, or “Program Pengelolaan Penyakit Kronis” (Prolanis) [8]. Prolanis is a system of governance of health services and health education that was designed for social health insurance members who suffer from hypertension and T2DM, to afford an optimal QoL independently [8].

Pharmacist counseling is an activity that aims to provide oral or written treatment information to patients regarding how to use the right medication by explaining the side effects of the drug, how it should be stored, and the recommended diet and lifestyle modifications [9]. In turn, the Regulation of the Minister of Health of the Republic of Indonesia (Permenkes RI) of 2016 states that pharmacist counseling is an activity that provides advice or suggestions related to drug therapy from a pharmacist (counselor) to patients and/or their families [10]. Concluded that pharmacist intervention was effective in achieving glycemic control and better QoL in patients with T2DM. In addition, other studies also concluded that patient counseling imparted by pharmacists improves the overall QoL in patients with diabetes [11].

Based on the 2014 report of the South Sulawesi Provincial Health Office, Makassar City exhibited the highest prevalence of T2DM in South Sulawesi Province [12]. Previously, we measured the QoL among Prolanis T2DM patients in four primary health care centers (Puskesmas) using the Bahasa Indonesia version of the European Quality of Life 5 Dimensions 5 Levels (EQ-5D-5 L) questionnaire. Prolanis T2DM patients in those Puskesmas exhibited a low QoL profile [13]. Therefore, in this study, pharmacist counseling was provided as an intervention to the patients with T2DM from Prolanis, followed by the analysis of the impact of these interventions on the HRQoL of these patients.

## Methods

### Study design and setting

This cluster randomized controlled trial was designed to include two groups (the control and intervention groups), with a pre- and a post-test procedure. Participants were Prolanis T2DM patients who attended four Puskesmas (Antang, Batua, Jongaya, and Tamalanrea) in Makassar City, South Sulawesi, Indonesia from August 2017 to August, 2018.

Cluster randomization was performed in this study. The four Puskesmas were randomized by asking the person in charge of the Prolanis at these centers to choose a closed envelope containing an identifier indicating the control group or the intervention group. Two Puskesmas were used as the control group and the remaining two were used as the intervention group.

Sociodemographic and clinical data (including medication profile and glycated hemoglobin (HbA1c) levels) were obtained from medical records and direct interviews. All patients provided written informed consent before participating in the study. The participants were administered the EQ-5D-5 L questionnaire in the first month of the study, as a pre-test procedure. The patients in the control group participated in the standard Prolanis T2DM program for 6 months and were asked to fill out the questionnaire again at the 6-month time point, as a post-test procedure. To control confounding of the results after 6 mo, the patients in the control group were given only the standard medicine information service by the pharmacists. Thus, each month all patients in both the groups met the pharmacists. The patients in the intervention group participated in the standard Prolanis T2DM program and received a 15 min face-to-face counseling session including the standard medicine information service from a pharmacist once a month for 6 months. At the 6-month time point, subjects in the intervention group were asked to fill out the EQ-5D-5 L questionnaire again, as a post-test procedure.

## Eligibility criteria

The inclusion criteria of this study were (1) registration in the Prolanis at BPJS Makassar City, (2) age between 18 and 65 years, (3) HbA1c level ≥ 6.5%, and (4) willingness to participate in research by signing an informed consent (for all T2DM patients with or without comorbidities). The exclusion criteria of this study were (1) irregular control schedules, (2) incomplete medical record data, and (3) circumstances that did not allow filling out the questionnaires (e.g., inability to speak, see, or hear).

### Ethics

The study protocol was approved by the Health Research Ethics Committee of the Faculty of Medicine, Hasanuddin University, Makassar City, Indonesia (No.146/H4.8.4.5.31/PP36-KOMETIK/2017) and was registered online through https://clinicaltrials.gov (ID:NCT04313829). Before data collection, the nature and the objectives of the study were explained to the patients who agreed to participate in the study and the confidentiality of the information was assured.

### Counselling training for pharmacists

To standardize the ability of pharmacists to provide counseling, an 8 h training was administered by two invited experts to the pharmacists who provided counseling at the four Puskesmas. All participants had a valid license and had a working period of more than 6 months. Participants were provided the counseling modules that consisted of techniques that are used to provide counseling in health care facilities.

### Instrument

We used the counseling module (in the form of a guide book) for pharmacist-based counseling that had been validated regarding constructive content by an endocrinologist and a pharmacist expert in diabetes drug counseling. The module explained the T2DM causes and symptoms, the reasons for the importance of therapy, the non-pharmacological and pharmacological therapies available (drug names, strengths, indications, rules of use, side effects, interactions, and storage), the purpose of controlling blood sugar levels, medications that need to be avoided, and guidelines for missed doses. The pharmacists explained all the contents within the module in 15 min to each patient of the intervention group for 6 mo. As an ethical consideration, after the study period was completed, patients in the control group were given the same explanation during counseling.

The QoL of Prolanis T2DM patients was measured using the Euro Quality of Life 5 Dimension 5 Level (EQ-5D-5 L) questionnaire. The EQ-5D-5 L is a generic HRQoL assessment tool that was obtained by filling out the registration form through https://euroqol.org/registrationform/. The EQ-5D-5 L questionnaire consists of two parts: a descriptive system and a Visual Analogue Scale (VAS). The descriptive system describes the health state and consists of five dimensions: mobility, self-care, usual activities, pain/discomfort, and anxiety/depression. Each state is attributed a 5-digit code. For example, a health state code of 11,111 indicates the absence of problems on all five dimensions; a health state code of 12,345 indicates no problems in the mobility dimension, slight problems in the self-care dimension, moderate problems in the usual activities dimension, and severe issues in the pain or discomfort with anxiety/depression dimension [14]. Patients who reported problems were then categorized into two categories, a “has no problem” category (health state code of 1) and “has problem” category (health state code of 2–5) for each level of each dimension [14]. Furthermore, the value of the health state code that was obtained from the descriptive system was converted using the index value of the EQ-5D-5 L Indonesian set value [15]. The second part of the EQ-5D-5 L questionnaire is the EQ-VAS, which is a thermometer-like scale (ranging from 0 to 100) that reflects the patient’s health in general [14]. EQ-VAS represents the patient perspective, where zero indicates the worst imaginable health state and 100 reflects the best imaginable health state [14].

### Statistical analysis

Statistical analysis was performed using the IBM SPSS Statistics for Windows software, version 22.0. (IBM Corp, Armonk, NY, USA). Descriptive statistics and chi-squared tests were used to investigate the distribution of variables among Prolanis T2DM patients. A Wilcoxon paired *t*-test was used to compare mean differences in health state code, patient-reported problems, EQ-5D-5 L index score, EQ-VAS score, and HbA1c at the 1- and 6-month time points. The Mann–Whitney independent *t*-test was used to compare differences between the two groups. Significance was set at *P* < 0.05.

## Results

### Participant recruitment process

**Fig. 1:**
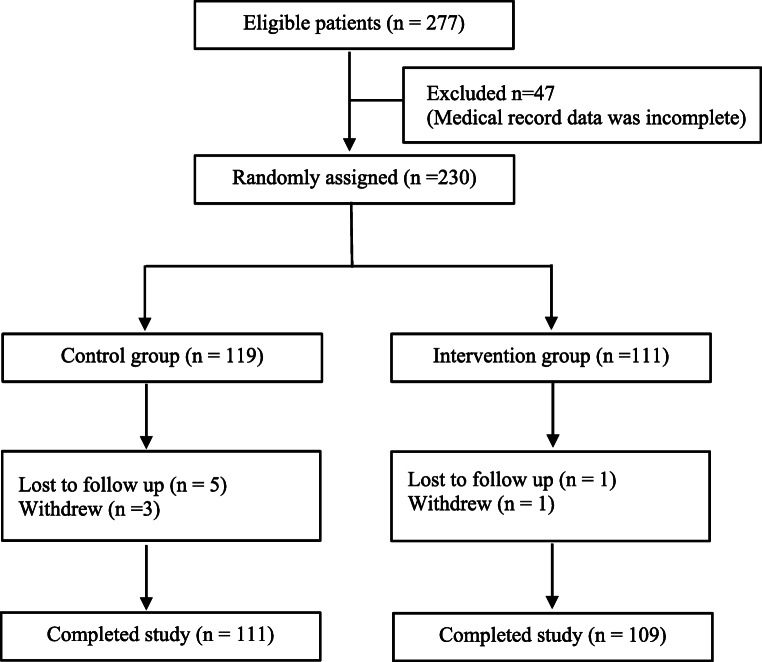
Flow chart of the participant recruitment process in accordance with the Consolidated Standards of Reporting Trials (CONSORT) guidelines)

Figure 1 depicts the participant recruitment Process. Among the 277 patients who were assessed for study eligibility, 47 individuals were excluded because of incomplete medical records. Thus, 230 patients were eligible for random group assignment based on the results of the Puskesmas randomization. The final number of patients who met the requirements for analysis was 220: 111 patients were assigned to the control group and 109 were assigned to the intervention group.

### Characteristic of the participants

Table 1 lists the sociodemographic and clinical characteristics of the study participants. The average age of the respondents was 57.71 ± 5.6 years (mean ± SD) and 64,09% of the subjects were women. Most patients had only graduated from elementary school (46.36%), were not working or were retired (72.27%), were married (88.18%), and had an average monthly household income above the regional minimum salary (53.18%). Most respondents were diagnosed with diabetes more than 5 years before participation in this study (51.82%) and 62.72% of the participants had a diagnosis of hypertension. There was a significant difference in the level of education between the control and the intervention groups (*P* = 0.039), which may be attributed to the geographical location of the Puskesmas; individuals in the control group resided relatively closer to the city center and had relatively higher education than intervention group.

**Table 1:** Sociodemographic characteristics and clinical condition

Characteristics	Control (n = 111)		Intervention (*n* = 109)		Total (*n* = 220) n (%)	*P* value
	n	(%)	n	(%)		
Gender						
Male	37	33,33	42	37,84	79 (35.91)	0,424
Female	74	66,67	67	60,36	141 (64.09)	
Age (mean 57.71 ± 5.6) years						
<50 years	15	13,51	5	4,50	20 (9.09)	
50–59 years	46	41,44	48	43,24	94 (42.73)	0,081
60–65 years	50	45,05	56	50,45	106 (48,18)	
Level of education						
Elementary school	45	40,54	57	51,35	102 (46,36)	
Junior high school	15	13,51	14	12,61	29 (13,18)	0,039^*^
Senior high school	27	24,32	25	22,52	52 (23,64)	
University	24	21,62	13	11,71	37 (16,82)	
Occupation						
Working	26	23,42	35	31,53	61 (27,73)	0,152
Not working/retired	85	76,58	74	66,67	159 (72,27)	
Marital status						
Married	56	50,45	98	88,29	194 (88,18)	0,434
Unmarried	55	49,55	11	9,91	26 (11,82)	
Monthly Income (IDR)						
<RMS^*^	96	86,49	61	54,95	117 (53,18)	0,415
>RMS	15	13,51	48	43,25	103 (46,82)	
Duration of diabetes						
≤5 years	58	52,25	48	43,24	106 (48,18)	0,225
>5 years	53	47,75	61	54,95	114 (51,82)	
Comorbidities						
Hyperlipidemia	16	14,41	23	20,72	39 (17.73)	
Hypertension	68	61,26	70	63,06	138 (62.72)	
Kidney failure	11	9,91	4	3,60	15 (6.82)	0,110
Heart failure	4	3,60	4	3,60	8 (3.64)	
No comorbidities	12	10,81	8	7,21	20 (9.09)	

Reporting rate: chi-squared test, *P* value <0.05

RMS, Regional Minimum Salary; IDR, Indonesian Rupiah

^*^Regional Minimum Salary of Makassar City: IDR 2.504.500

^*^Significant

Table 2 shows the distribution of antidiabetics between two groups. The three types of antidiabetic drugs given to the patients included metformin, acarbose, and glimepiride. The distribution of antidiabetics between the two groups was similar and were assumed to have the same profile.

**Table 2:** Distribution of antidiabetics between the two groups

No.	Medicines	Control group		Intervention group	
		n (patients)	%	n (patients)	%
1	Metformin 500 mg	56	50,45	51	46,79
2	Acarbose 50 mg	10	9,00	8	7,34
3	Glimepiride 1 mg	15	13,51	20	18,35
4	Glimepiride 2 mg	5	4,51	3	2,75
5	Glimepiride 3 mg	5	4,51	4	3,67
6	Glimepiride 4 mg	20	18,02	23	21,10
Total		111	100	109	100

### Profile of QoL and clinical outcomes

Tables 2 and 3 illustrates the health state codes of the participants, patient-reported problems, EQ-5D-5 L index scores, VAS scores, and clinical outcomes before and after the pharmacist counseling intervention. Regarding the health state code of the patients in the intervention group, three health state dimensions, i.e., mobility, self-care, and pain/discomfort, exhibited significant changes after the intervention, (*P* = 0.000. *P* = 0.002, and *> P* = 0.025 respectively). In contrast, patients in the control group showed significant changes only in the dimension of pain/discomfort after the intervention (*P* = 0.008). The comparison of the changes in the average health state score (from before to after the intervention) between the two groups revealed a significant difference in the dimensions of self-care (*P* = 0.005) and anxiety/depression (*P* = 0.000).

**Table 3:** Profile of quality of life and clinical outcomes

Health state dimensions	Control group (n = 111)				Intervention group (n = 109)				
	1 month	6 months	Δ change	*P* value	1 month	6 months	Δ change	*P* value	*P* value
Mobility	2,15 ± 0,87	2,08 ± 0,89	0,07 ± 0,40	0,059	2,02 ± 0,79	1,84 ± 0,72	0,17 ± 0,40	0,000^*^	0,060
Self-care	2,08 ± 0,96	2,11 ± 0,94	-0,03 ± 0,34	0,408	1,92 ± 0,82	1,82 ± 0,80	0,10 ± 0,33	0,002^*^	0,005^*^
Usual activities	2,10 ± 0,85	2,08 ± 0,84	0,02 ± 0,30	0,530	2,02 ± 0,86	1,97 ± 0,82	0,05 ± 0,25	0,058	0,457
Pain/discomfort	2,05 ± 0,94	1,99 ± 0,94	0,06 ± 0,24	0,008^*^	2,01 ± 0,87	1,96 ± 0,85	0,05 ± 0,21	0,025^*^	0,577
Anxiety/depression	2,10 ± 0,97	2,11 ± 0,95	-0,01 ± 0,25	0,707	2,05 ± 0,85	1,91 ± 0,80	0,06 ± 0,83	0,008	0,000^*^
Mobility	85 (76,58%)	81 (72,97%)	4 (3,61%)	0,109	89 (81,65%)	81 (74,31%)	8 (7,34%)	0,011^*^	0,603
Self-care	75 (67,57%)	78 (70,27%)	−3 (−2,7%)	0,259	78 (71,56%)	73 (66,97%)	5 (4,59%)	0,058	0,212
Usual activities	80 (72,07%)	80 (72,07%)	0 (0%)	1000	83 (76,15%)	82 (75,23%)	1 (0,92%)	0,566	0,388
Pain/discomfort	71 (63,96%)	67 (60,36%)	4 (3,60%)	0,045^*^	81 (74,31%)	80 (73,40%)	1 (0,91%)	0,320	0,644
Anxiety/depression	75 (67,57%)	77 (69,37%)	−2 (−1,80%)	0,417	86 (78,90%)	71 (64,14%)	15 (14,76%)	0,025^*^	0,001^*^
Index value	0,54 ± 0,32	0,55 ± 0,33	0,01 ± 0,09	0,192	0,59 ± 0,28	0,63 ± 0,26	0,04 ± 0,09	0,000^*^	0,041^*^
VAS score	70,73 ± 15,00	70,66 ± 15,19	−0,07 ± 4,35	0,862	74,21 ± 14,34	77,01 ± 13,62	2,66 ± 5,39	0,000^*^	0,000^*^
HbA1c level	8,90 ± 1,94	9,32 ± 1,99	−0,42 ± 0,92	0,000^*^	8,45 ± 1,76	7,94 ± 1,41	0,51 ± 0,62	0,000^*^	0,000^*^

^*^Significant

Patients in the control group reported a significant improvement only in the pain/discomfort dimension (*P* = 0.045), whereas patients in the intervention group experienced improvement in two dimensions after the intervention, the mobility (*P* = 0.011) and the anxiety/depression (*P* = 0.025) dimensions, at the 6-month time point. The comparison of the changes in the patient-reported problem score (from before to after the intervention) between the two groups revealed a significant difference in the dimension of anxiety/depression (*P* = 0.001).

The QoL of the intervention group after 6 months of pharmacist counseling was increased compared with that observed for the control group at the 6-mpnth evaluation (EQ-5D-5 L index score, 0.63 vs. 0.55, *P* = 0.000; and EQ-VAS score, 77.01 vs. 70.66, *P* = 0.000). The increase in QoL detected in patients in the intervention group was consistent with the improvement in their clinical outcome, as assessed based on the significant decrease in the levels of HbA1c (from 8.45% to 7.94%; *P* = 0.000). In contrast, the HbA1c levels in the control group increased from 8.90% to 9.32% (*P* = 0.000). The comparison of the changes (from before to after the intervention) in the mean EQ-5D-5 L index score, EQ-VAS score, and HbA1c levels between the two groups revealed a significant difference in the EQ-5D-5 L index score (*P* = 0.041), EQ-VAS score (*P* = 0.000), and HbA1c level (*P* = 0.000); this implies that pharmacist counseling interventions were effective in improving the QoL and clinical outcomes of Prolanis T2DM patients.

## Discussion

The present study showed that the pharmacist counseling intervention significantly improved the HRQoL of Prolanis T2DM patients by increasing the EQ-5D-5 L index score and the EQ-VAS score and decreasing HbA1c levels. The overall HRQoL of the patients in the intervention group was significantly improved at 6 mo compared with the control group. The results of this study are consistent with the research reported in India by Prasanth (2018), who concluded that counseling provided by clinical pharmacists can be very beneficial for the management and monitoring of patients with metabolic syndrome, to improve their QoL [16]. Moreover, a study of patients with T2DM undertaken at a military hospital in Myanmar proved that pharmacist intervention increased significantly the QOL of these patients compared with those who did not receive the intervention [17].

In this study, there was a significant difference in the level of education between the control and intervention groups. The control group tended to have higher education than intervention group, which may be attributed to the geographical location of the Puskesmas (individuals in the control group resided relatively closer to the city center). However, the QoL of the intervention group was better than the control group. This revealed that education in the intervention group played a role in increasing QoL. A study in Iran showed that education to patients with diabetes could improve HbA1c, behavior modification, and HRQoL, [18] and a systematic review in 2012 revealed that self-management education in people with T2DM resulted in improvements in clinical, lifestyle, and psychosocial outcomes [19].

In this study, patients who received the intervention experienced a significant improvement in the dimension of self-care. This might be attributed to the counseling material about the self-management of symptoms, treatment, and lifestyle changes in patients with T2DM. This finding confirms the result of another study that reported that patient who participated in medical appointments was associated with better understanding of self-care [20]. Moreover, the dimensions of mobility and pain/discomfort were also improved in the intervention group. The dimension of mobility was the most widely reported patient-reported problem in the two groups included in this study. This might be explained by the fact that 106 patients who participated in this study were more than 60 years old.

In addition to the mobility dimension, the anxiety/depression dimension was also improved in the intervention group. This is in line with the results of the study reported by Scott et al. who described an increase in QoL and improvement in the anxiety dimensions of diabetic patients managed by pharmacists [21]. In another study performed over a period of 12 months, the mental component summary (MCS) of the QoL questionnaire was improved after the pharmacist intervention, which did not seem to affect the physical component [22].

The QoL of patients with T2DM can vary individually, as it can be affected by disease factors and/or the specific treatment administered to each patient [23]. Several studies have acknowledged the importance of pharmacists in providing counseling to diabetic patients [24, 25]. Pharmacists play an important role in this setting by providing counseling with a demonstrated positive impact on healthcare, as a significant improvement in the QoL of diabetic patients has been observed after this type of intervention [26]. In our study, the EQ-5D-5 L index score and the VAS score recorded in the intervention group after the intervention of pharmacists were increased by 0.04 (*P* = 0.041) and 2.66 (*P* = 0.000) points, respectively, whereas they were decreased by 0.01 (*P* = 0.192) and − 0.07 (*P* = 0.862) points, respectively, in the control group. These results suggest that the QoL of patients with T2DM is improved after a counseling intervention by a pharmacist. Our findings are in line with those of the Kjeldsen study, which reported an increase in the EQ-5D-5 L index score of 0.06 in a pharmacist-intervened group of patients with T2DM [27]. Another study showed a significant increase (*P* = 0.007) in the VAS score in a group of patients with T2DM that received a pharmacist intervention for 6 months (Butt, 2015). In addition, Krass et al. reported a change in QoL between the intervention and control groups, of 0.057 and − 0.02, respectively (*P* = 0.02).

Together with the increase in the EQ-5D-5 L index score and EQ-VAS score, the HbA1c levels were decreased in the intervention group after the pharmacist counseling intervention, which was in contrast with the change in HbA1c levels detected in the control group. The glycated hemoglobin, hemoglobin A1c or HbA1c test represents the average level of blood sugar over the past two to 3 mo [28]. The study by Nichols [29] revealed that the increase in HbA1c was associated with non-adherence in patients with diabetes mellitus. In our study, the control group had increased HbA1c, which was assumed that patients had lower adherence than the intervention group. This suggests that pharmacist interventions improve the clinical outcomes of, and patient compliance to, T2DM therapy. Moreover, a meta-analysis showed that self-management interventions led by pharmacists decreased the levels of HbA1c in diabetic patients [30]. Finally, other research also demonstrated that the QoL of patients can be affected by adherence to anti-diabetic treatment and by decreasing the levels of HbA1c [31].

This study had a limitation in data collection. We only collected data from four of the 40 Puskesmas located in Makassar city. Although the representativeness of the study sample regarding Makassar city could not be claimed directly, a report from BPJS health data of the Makassar City in December 2016 stated that the four Puskesmas at which our study was conducted were those with the highest number of registered Prolanis patients in Makassar city (277 out of 1185 patients).

## Conclusion

The pharmacist counseling intervention affected the HRQoL of Prolanis T2DM patients by increasing the EQ-5D-5 L index score and the EQ-VAS score and decreasing HbA1c levels. Therefore, the inclusion of pharmacist counseling in the management of patients with T2DM from the Prolanis initiative of the BPJS system of Indonesia should be considered.

## Electronic supplementary material

ESM 1 (PDF 82 kb)
