# Supplementary material for: Impact of pharmacist counseling on health-related quality of life of patients with type 2 diabetes mellitus: a cluster randomized controlled study
Source: J Diabetes Metab Disord. 2020 Jun 3;19(2):675–82. doi: 10.1007/s40200-020-00528-x (PMC7843751; doi:10.1007/s40200-020-00528-x)
Supplement: Supplementary file 1 — (PDF 82 kb) [file 40200_2020_528_MOESM1_ESM.pdf]

**ClinicalTrials.gov Protocol Registration and Results System (PRS) Receipt**
Release Date: March 19, 2020

**ClinicalTrials.gov ID: NCT04313829**

## Study Identification

Unique Protocol ID: DM-202003.01

Brief Title: Pharmacist Counseling on the Health-related Quality of Life of Patients With Type 2 Diabetes Mellitus

Official Title: Impact of Pharmacist Counseling on the Health-related Quality of Life of Patients With Type 2 Diabetes Mellitus: a Cluster Randomized Controlled Study

Secondary IDs:

## Study Status

Record Verification: March 2020

Overall Status: Completed

Study Start: August 1, 2017 [Actual]

Primary Completion: February 1, 2018 [Actual]

Study Completion: August 31, 2018 [Actual]

## Sponsor/Collaborators

Sponsor: Universitas Padjadjaran

Responsible Party: Sponsor

Collaborators: Ministry of Research, Technology and Higher Education of the Republic of Indonesia

## Oversight

U.S. FDA-regulated Drug: No

U.S. FDA-regulated Device: No

U.S. FDA IND/IDE: No

Human Subjects Review: Board Status: Approved
Approval Number: No. 146/H4.8.4.5.31/PP36-KOMETI
Board Name: Health Research Ethics Committee of the Faculty of Medicine
Board Affiliation: Hasanuddin University
Phone: +62 81241850858
Email:
Address:

Lantai 3 Gedung Laboratorium Terpadu
Jl. Perintis Kemerdekaan Kampus Tamalanrea Km. 10 Makassar, 90245

Data Monitoring:

## Study Description

Brief Summary: The quality of life (QoL) of patients with type 2 diabetes mellitus (T2DM) is a measure of the successful outcomes of therapy. The program of management of chronic diseases “Program Pengelolaan Penyakit Kronis” (Prolanis) among patients with hypertension and T2DM is a new strategy of the Badan Penyelenggara Jaminan Sosial (BPJS), which is the Indonesian national health insurance system. The impact of pharmacist counseling interventions on health-related QoL (HRQoL) was analyzed in Prolanis T2DM patients.

Detailed Description: A cluster randomized controlled trial that was designed to include two groups (control (n = 111) and intervention (n = 109) groups), and pre- and post-test procedures were conducted. The participants were Prolanis T2DM patients who attended four primary health-care centers (Puskesmas) in Makassar City, South Sulawesi, Indonesia from August 2017 to August 2018. The intervention group received systematic counseling for 6 months. The data were collected using the Bahasa Indonesia version of the European Quality of Life 5 Dimensions 5 Levels (EQ-5D-5L) questionnaire and were analyzed using EQ-5D preference weight for each health state with the Indonesian EQ-5D-5L value Set. Furthermore, the EQ-5D index and the EQ-5D VAS score were calculated and HbA1c levels were assessed.

## Conditions

Conditions: Type 2 Diabetes Mellitus

Keywords:

## Study Design

Study Type: Interventional

Primary Purpose: Supportive Care

Study Phase: N/A

Interventional Study Model: Parallel Assignment
All patients provided written informed consent before participating in the study. The participants were administered the EQ-5D-5L questionnaire in the first month of the study, as a pre-test procedure. The patients in the control group participated in the standard Prolanis T2DM program for 6 months and were asked to fill out the questionnaire again at the 6-month time point, as a post-test procedure. The patients in the intervention group participated in the standard Prolanis T2DM program and received a 15 min face-to-face counseling session from a pharmacist once a month for 6 months. At the 6-month time point, subjects in the intervention group were asked to fill out the EQ-5D-5L questionnaire again, as a post-test procedure. Patients in the control group also received a 15 min face-to-face counseling session from a pharmacist once a month for 6 months after the intervention study was completed.

Number of Arms: 2

Masking: Single (Care Provider)
Cluster randomization by institution was used in this study. The four Puskesmas were randomized by asking the person in charge of the Prolanis at these centers to choose a closed envelope containing an identifier indicating the

control group or the intervention group. Two Puskesmas were used as the control group and the remaining two were used as the intervention group.

Allocation: Randomized

Enrollment: 220 [Actual]

## Arms and Interventions

| Arms | Assigned Interventions |
|---|---|
| Active Comparator: Intervention group<br>The intervention group received Pharmacist counseling for 15 minutes include giving standard medicine information service and explaining the validated pharmacist counseling module which contained the T2DM causes and symptoms, the reasons for the importance of therapy, the non-pharmacological and pharmacological therapies available (drug names, strengths, indications, rules of use, side effects, interactions, and storage), the purpose of controlling blood sugar levels, medications that need to be avoided, and guidelines for missed dose. | Behavioral: Pharmacist Counseling Intervention<br>We used the counseling module (in the form of a guide book) for pharmacist-based counseling that had been validated regarding constructive content by an endocrinologist and a pharmacist expert in diabetes drug counseling. The module explained the T2DM causes and symptoms, the reasons for the importance of therapy, the non-pharmacological and pharmacological therapies available (drug names, strengths, indications, rules of use, side effects, interactions, and storage), the purpose of controlling blood sugar levels, medications that need to be avoided, and guidelines for missed doses. The pharmacists should explain all the content within the module in 15 minutes to each patient of the intervention group each month for 6 months. As an ethical consideration, the control group patients were given the same explanation module through pharmacist counseling after the study finished. |
| No Intervention: Control group<br>The control group received standard medicine information services by Pharmacists. | |

## Outcome Measures

Primary Outcome Measure:

1. Measurement of Quality of Life (QoL) using the Euro Quality of Life 5 Dimension 5 Level (EQ-5D-5L) questionnaire
   The QoL of Prolanis T2DM patients was measured using the Euro Quality of Life 5 Dimension 5 Level (EQ-5D-5L) questionnaire.

   The EQ-5D-5L questionnaire consists of two parts: a descriptive system and a Visual Analogue Scale (VAS).

   The descriptive system describes the health state and consists of five dimensions: mobility, self-care, usual activities, pain/discomfort, and anxiety/depression. Patients who reported problems were then categorized into two categories, a “has no problem” category (health state code of 1) and “has problem” category (health state code of 2–5) for each level of each dimension.

   The second part of the EQ-5D-5L questionnaire is the EQ-VAS, which is a thermometer-like scale (ranging from 0 to 100) that reflects the patient’s health in general. EQ-VAS represents the patient perspective, where zero indicates the worst imaginable health state and 100 reflects the best imaginable health state.

   [Time Frame: 6 months]

Secondary Outcome Measure:

2. Measurement of HbA1c
   Measurement of HbA1c was done over a period of 2-4 months. In addition, HbA1c was also be considered compatible with therapy.

   [Time Frame: 6 months]

## Eligibility

Minimum Age: 18 Years

Maximum Age: 65 Years

Sex: All

Gender Based: No

Accepts Healthy Volunteers: No

Criteria: Inclusion Criteria:

- Registration in the Prolanis at BPJS Makassar City,
- Age between 20 and 65 years,
- HbA1c level ≥6.5%, and
- Willingness to participate in research by signing an informed consent (for all T2DM patients with or without comorbidities)

Exclusion Criteria:

- Irregular control schedules,
- Incomplete medical record data,
- Circumstances that did not allow filling out the questionnaires (e.g., inability to speak, see, or hear)

## Contacts/Locations

Central Contact Person: Fajriansyah Fajriansyah, Master of Sciences
Telephone: +62 85299903000
Email: fajriansyah.fajrin@yahoo.com

Central Contact Backup:

Study Officials: Fajriansyah Fajriansyah, Master
Study Principal Investigator
Faculty of Pharmacy Universitas Padjadjaran Bandung

Locations: **Indonesia**

Puskesmas Tamalanrea
Makassar, South Sulawesi, Indonesia
Contact: Asrina , Pharmacist +62 85256618209
Contact: A. Erny , Dentist +62411 582289

Puskesmas Batua
Makassar, South Sulawesi, Indonesia
Contact: Decy Tandilintin, Pharmacist +62 8124238300
Contact: Ramluddin , Nurse +62411 493808

Puskesmas Jongaya
Makassar, South Sulawesi, Indonesia
Contact: Latifah , Pharmacist +62 81321507678
Contact: Hatase Nurna, Doctor +62411 867406

Puskesmas Antang
Makassar, South Sulawesi, Indonesia
Contact: Tenriani , Pharmacist +62 85343881122
Contact: Masni , Public health +62411 494031

## IPDSharing

Plan to Share IPD: No

## References

Citations:

Purba FD, Hunfeld JAM, Iskandarsyah A, Fitriana TS, Sadarjoen SS, Ramos-Goñi JM, Passchier J, Busschbach JJV. The Indonesian EQ-5D-5L Value Set. Pharmacoeconomics. 2017 Nov;35(11):1153-1165. doi: 10.1007/s40273-017-0538-9. PubMed 28695543

Agborsangaya CB, Lau D, Lahtinen M, Cooke T, Johnson JA. Health-related quality of life and healthcare utilization in multimorbidity: results of a cross-sectional survey. Qual Life Res. 2013 May;22(4):791-9. doi: 10.1007/s11136-012-0214-7. Epub 2012 Jun 9. PubMed 22684529

Herdman M, Gudex C, Lloyd A, Janssen M, Kind P, Parkin D, Bonsel G, Badia X. Development and preliminary testing of the new five-level version of EQ-5D (EQ-5D-5L). Qual Life Res. 2011 Dec;20(10):1727-36. doi: 10.1007/s11136-011-9903-x. Epub 2011 Apr 9. PubMed 21479777

**[Study Results]** Steinsbekk A, Rygg LØ, Lisulo M, Rise MB, Fretheim A. Group based diabetes self-management education compared to routine treatment for people with type 2 diabetes mellitus. A systematic review with meta-analysis. BMC Health Serv Res. 2012 Jul 23;12:213. doi: 10.1186/1472-6963-12-213. Review. PubMed 22824531

**[Study Results]** Megari K. Quality of Life in Chronic Disease Patients. Health Psychol Res. 2013 Sep 23;1(3):e27. doi: 10.4081/hpr.2013.e27. eCollection 2013 Sep 24. Review. PubMed 26973912

**[Study Results]** Kjeldsen LJ, Bjerrum L, Dam P, Larsen BO, Rossing C, Søndergaard B, Herborg H. Safe and effective use of medicines for patients with type 2 diabetes - A randomized controlled trial of two interventions delivered by local pharmacies. Res Social Adm Pharm. 2015 Jan-Feb;11(1):47-62. doi: 10.1016/j.sapharm.2014.03.003. Epub 2014 Apr 12. PubMed 24798710

**[Study Results]** Nichols GA, Rosales AG, Kimes TM, Tunceli K, Kurtyka K, Mavros P. The Change in HbA1c Associated with Initial Adherence and Subsequent Change in Adherence among Diabetes Patients Newly Initiating Metformin Therapy. J Diabetes Res. 2016;2016:9687815. doi: 10.1155/2016/9687815. Epub 2016 Aug 7. PubMed 27579326

**[Study Results]** van Eikenhorst L, Taxis K, van Dijk L, de Gier H. Pharmacist-Led Self-management Interventions to Improve Diabetes Outcomes. A Systematic Literature Review and Meta-Analysis. Front Pharmacol. 2017 Dec 14;8:891. doi: 10.3389/fphar.2017.00891. eCollection 2017. Review. PubMed 29311916

**[Study Results]** Gusmai Lde F, Novato Tde S, Nogueira Lde S. [The influence of quality of life in treatment adherence of diabetic patients: a systematic review]. Rev Esc Enferm USP. 2015 Oct;49(5):839-46. doi: 10.1590/S0080-623420150000500019. Review. Portuguese. PubMed 26516756

Links:

Available IPD/Information:
